# Analysis of the incidence and risk factors of blood transfusion in total knee revision: a retrospective nationwide inpatient sample database study

**DOI:** 10.1186/s12891-024-07331-2

**Published:** 2024-03-20

**Authors:** Xiaoyin Li, Hao Xie, Shuxia Liu, Jian Wang, Zhanjun Shi, Qiaobing Yao, Qinfeng Yang, Qiuhong Li, Liangxiao Bao

**Affiliations:** 1 Division of Orthopaedic Surgery, Department of Orthopaedics, Nanfang Hospital, Southern Medical University, Guangzhou, 510515 China; 2 Division of Orthopaedics and Traumatology, Department of Orthopaedics, Nanfang Hospital, Southern Medical University, Guangzhou, 510515 China

**Keywords:** Blood transfusion, Total knee revision, Risk factors, Incidence, Nationwide inpatient sample

## Abstract

**Objective:**

This study sought to determine the incidence and risk factors of blood transfusion among patients undergoing total knee revision (TKR) using a nationwide database.

**Methods:**

A retrospective data analysis was conducted based on the Nationwide Inpatient Sample (NIS), enrolling patients who underwent TKR from 2010 to 2019 with complete information. The patients were divided into two groups based on whether they received blood transfusion or not. The demographic characteristics (race, sex, and age), length of stay (LOS), total charge of hospitalization, hospital characteristics (admission type, insurance type, bed size, teaching status, location, and region of hospital), hospital mortality, comorbidities, and perioperative complications were analyzed. Finally, we conducted univariate and multivariate logistic regression to identify factors that were associated with TKR patients to require blood transfusion.

**Results:**

The NIS database included 115,072 patients who underwent TKR. Among them, 14,899 patients received blood transfusion, and the incidence of blood transfusion was 13.0%. There was a dramatic decrease in the incidence over the years from 2010 to 2019, dropping from 20.4 to 6.5%. TKR patients requiring transfusions had experienced longer LOS, incurred higher total medical expenses, utilized Medicare more frequently, and had increased in-hospital mortality rates (all *P* < 0.001). Independent predictors for blood transfusion included advanced age, female gender, iron-deficiency anemia, rheumatoid disease, chronic blood loss anemia, congestive heart failure, coagulopathy, uncomplicated diabetes, lymphoma, fluid and electrolyte disorders, metastatic carcinoma, other neurological diseases, paralysis, peripheral vascular disorders, pulmonary circulation disorders, renal failure, valvular disease, and weight loss. In addition, risk factors for transfusion in TKR surgery included sepsis, acute myocardial infarction, deep vein thrombosis, pulmonary embolism, gastrointestinal bleeding, heart failure, renal insufficiency, pneumonia, wound infection, lower limb nerve injury, hemorrhage/seroma/hematoma, wound rupture/non healing, urinary tract infection, acute renal failure, and postoperative delirium.

**Conclusions:**

Our findings highlight the importance of recognizing the risk factors of blood transfusion in TKR to reduce the occurrence of adverse events.

**Supplementary Information:**

The online version contains supplementary material available at 10.1186/s12891-024-07331-2.

## Introduction

Total knee arthroplasty (TKA) is a widely practiced and effective approach for treating advanced knee joint diseases in clinical settings [1, 2]. Although the therapeutic effect of total knee arthroplasty has been established, complications, including periprosthetic joint infection, prosthesis loosening, dislocation, periprosthetic fracture, and pain can lead to the failure of primary joint replacement and demand of revision treatment [3–5]. Total knee revision (TKR) requires the removal of the previously replaced prosthesis and re-implantation of the new prosthesis. Approximately over 100,000 TKR procedures are carried out annually in the United States [6].

TKR surgery is intricate and often results in significant blood loss. Consequently, the need for blood transfusions during TKR is more frequent. Nevertheless, blood transfusions come with potential risks, including transfusion reactions, infections, immunosuppression, and surgical site infections [3]. Furthermore, blood transfusion increases medical resources consumption, length of stay (LOS), hospitalization expenses, complications, and even death [7, 8]. Hence, it is crucial to identify how often patients undergoing TKR require blood transfusions and the factors that increase this risk to minimize the necessity for blood transfusions.

Reports indicate that the occurrence of blood transfusions in total knee arthroplasty varies between 3.2% and 18.1%, while for TKR, it ranges from 9.8 to 19.8% from 2010 to 2015 [9]. The preoperative lower level of hemoglobin (Hb), female, extended operative duration and increased blood loss during surgery have been reported to be independently associated with blood transfusion in total joint arthroplasty (TJA) [10, 11]. However, no study has hitherto examined the occurrence and risk factors of blood transfusion in patients undergoing TKR with a substantial sample size. Therefore, this study aims to explore the frequency and factors contributing to blood transfusions following TKR, harnessing data from a nationwide database.

## Materials and methods

### Data source

This study drew upon data from the Nationwide Inpatient Sample (NIS) database, a component of the Healthcare Cost and Utilization Project facilitated by the Agency for Healthcare Research and Quality. In the United States, NIS stands out as the most extensive all-payer database encompassing hospital admissions. Sampling from over 1000 hospitals, NIS gathers data from about 20% of annual hospitalizations in the country [12–14]. Data for this study were extracted from the database, encompassing patient demographics (age, sex, and race), hospital characteristics (insurance type, admission type, hospital bed size, teaching status, location, and region), LOS, economic indicators, and diagnostic and procedural codes derived from the International Classification of Diseases (ninth and tenth revisions) Clinical Modification (ICD-9-CM and ICD-10-CM). This research study did not require approval from an ethics board since it used anonymous data that is publicly accessible.

### Data collection

**Fig. 1:**
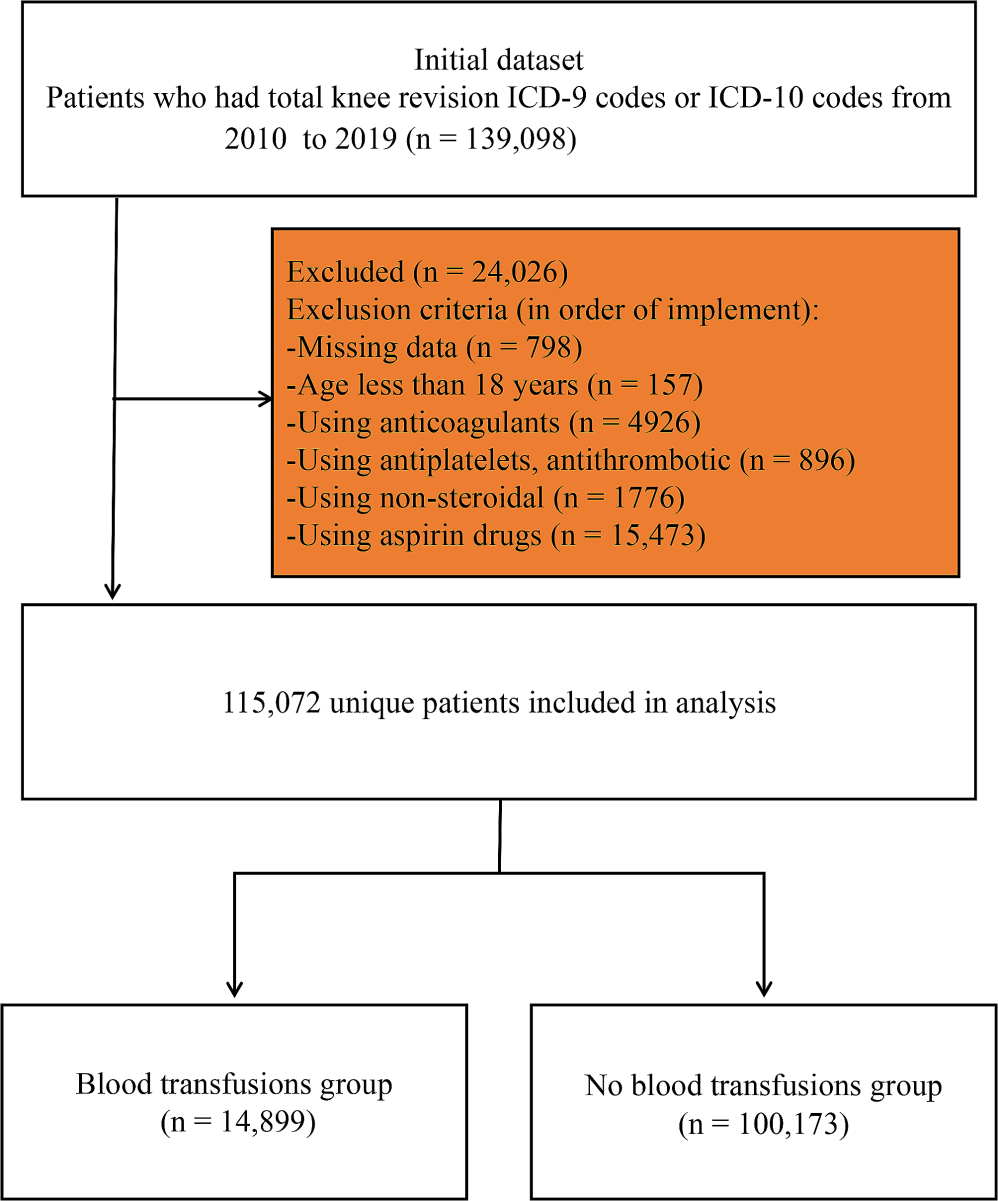
Exclusion process for patients undergoing total knee revision with blood transfusion

Data spanning from 2010 to 2019 was collected from the Nationwide Inpatient Sample database. The International Classification of Diseases, Ninth Revision, Clinical Modification (ICD-9-CM) and The International Classification of Diseases, tenth Revision, Clinical Modification primary procedure coding ICD-10-CM) indicative of total knee revision were used (Supplement 1) [6].The occurrence of blood transfusion was determined based on ICD-9-CM or ICD-10-CM diagnostic codes. Patients under 18 years old and those using anticoagulants, antiplatelets, antithrombotic, non-steroidal, and aspirin drugs were excluded (Fig. 1).

The patients included in the study were separated into two groups based on whether they received a blood transfusion. We evaluated patient demographics, hospital characteristics, and outcome measures such as LOS, economic indicators, and in-hospital mortality. Information about preoperative comorbidities and perioperative complications was extracted from the database using diagnostic codes from ICD-9-CM and ICD-10-CM (Table 1). Perioperative complications included: sepsis, acute myocardial infarction, deep vein thrombosis, convulsion, pulmonary embolism, gastrointestinal bleeding, heart failure, renal sufficiency, pneumonia, respiratory disease, urinary tract infection, acute renal failure and postoperative delirium, wound infection, lower limb nerve injury, hemorrhage/seroma/hematoma, wound rupture/non healing.

**Table 1:** Variables used in binary logistic regression analysis

Variables categories	Specific variables
**Patient demographics**	Age (≤ 50 years/ 51–60 years/ 61–70 years and ≥ 71 years), sex (male and female), race (White, Black, Hispanic, Asian or Pacific Islander, Native American and Other)
**Hospital characteristics**	Type of admission (non-elective, elective), bed size of hospital (small, medium, large), teaching status of hospital (nonteaching, teaching), location of hospital (rural, urban), type of insurance (Medicare, Medicaid, private insurance, self-pay, no charge, other), region of the hospital (northeast, Midwest or north central, south, west)
**Comorbidities**	AIDS, alcohol abuse, deficiency anemia, rheumatoid diseases, chronic blood loss anemia, congestive heart failure, chronic pulmonary disease, coagulopathy, depression, diabetes (uncomplicated), diabetes (with chronic complications), drug abuse, hypertension, hypothyroidism, liver disease, lymphoma, fluid and electrolyte disorders, metastatic cancer, neurological disorders, obesity, paralysis, peripheral vascular disorders, psychoses, pulmonary circulation disorders, renal failure, solid tumor without metastasis, peptic ulcer disease, valvular disease and weight loss

AIDS: Acquired immunodeficiency syndrome

### Data analysis

Analysis was conducted using Statistical Package for the Social Sciences (SPSS) 25.0 statistical software. Independent t-tests were applied to assess continuous data, while chi-square tests were used for comparing categorical data. Logistic regression analysis was employed to identify potential risk factors associated with blood transfusion. The regression analysis included all variables from NIS, covering patient demographics, hospital characteristics, and comorbidities (Table 1). Odds ratios (OR) and 95% confidence intervals (CI) were calculated after the logistic regression. Given the substantial sample size, statistical significance was considered for p-values less than 0.001.

## Results

### Incidence of blood transfusion after TKR

**Fig. 2:**
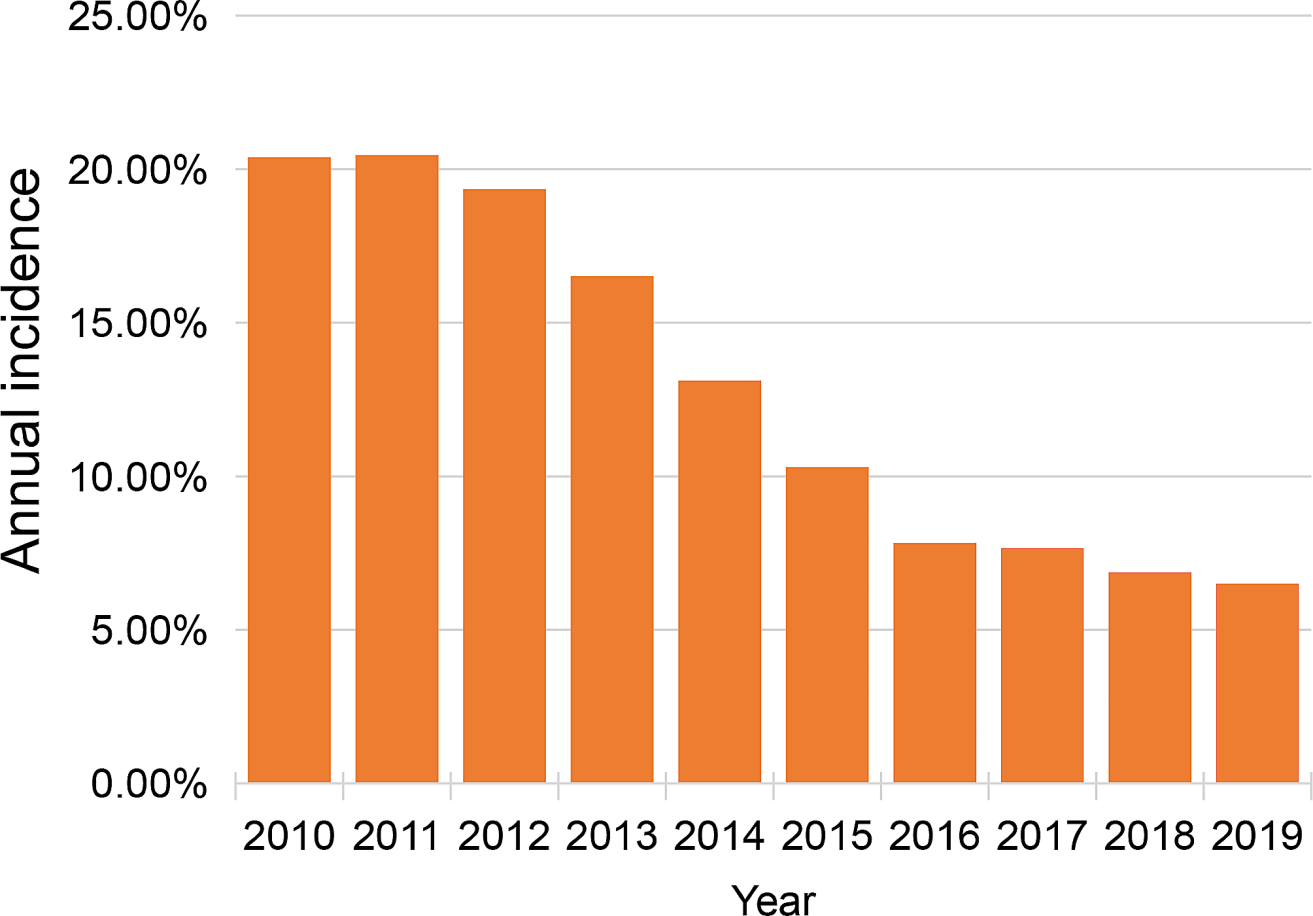
Annual incidence of blood transfusion in total knee revision

From 2010 to 2019, a total of 139,098 TKR cases were identified in the NIS database. After excluding the patients who did not meet the criteria, there were 115,072 patients undergoing TKR, among which 14,899 patients received blood transfusion, and the incidence of blood transfusion was 13.0% (Table 2). The data revealed a gradual decrease in the incidence of blood transfusion each year from 2011 to 2019, dropping from 20.4 to 6.5% (Fig. 2).

**Table 2:** Patient characteristics and outcomes after total knee revision(2010–2019)

Characteristics	Transfusion	No transfusion	*P*
**Total (n = count)**	14,899	100,173	
**Total incidence (%)**	13.0		
**Age (mean, SD)**	67.4 (11.4)	64.7(10.7)	< 0.001
**Age group (%)**			
≤ 50	1003 (6.7%)	8985 (9.0%)	< 0.001
51–60	2963 (19.9%)	25,593(25.5%)	
61–70	40,149 (32.0%)	35,378 (35.3%)	
≥ 71	6162 (41.4%)	30.2 (30.2%)	
**Gender (%)**			
Male	5348 (35.9%)	42,848 (42.8%)	< 0.001
Female	9553 (64.1%)	57,323 (57.2%)	
**Race (%)**			
White	10,549 (70.8%)	75,117 (75.0%)	< 0.001
Black	2084 (14.0%)	10,343 (10.3%)	
Hispanic	970 (6.5%)	5071 (5.1%)	
Asian or Pacific Islander	133 (0.9%)	722 (0.7%)	
Native American	81 (0.5%)	536 (0.5%)	
Other	1082 (7.3%)	8384 (8.4%)	
**Elixhauser score**			
0	776 (5.2%)	11,834 (11.8%)	< 0.001
1	1932 (13.0%)	22,310 (22.3%)	
≥ 2	12,191 (81.8%)	66,029(65.9%)	
**LOS (mean, SD)**	6.9 (6.8)	3.8 (4.1)	< 0.001
**TOTCHG (mean,$)**	121,039.0 (131905.6)	86,308.5 (72784.4)	< 0.001
**Type of insure (%)**			
Medicare	10,038 (67.4%)	56,571 (56.5%)	< 0.001
Medicaid	833 (5.6%)	5303(5.3%)	
Private insurance	3459 (23.2%)	32,303(32.2%)	
Self-pay	80 (0.5%)	570 (0.6%)	
No charge	12 (0.1%)	63 (0.1%)	
Other	477 (3.2%)	5363 (5.4%)	
**Bed size of hospital (%)**			
Small	2987 (20.0%)	23,759 (23.7%)	< 0.001
Medium	4097 (27.5%)	27,046 (27.0%)	
Large	7815 (52.5%)	49,368 (49.3%)	
**Elective admission (%)**	10,382 (69.7%)	83,523 (83.4%)	< 0.001
**Type of hospital (teaching, %)**	8686 (58.3%)	61,887 (61.8%)	< 0.001
**Location of hospital (urban, %)**	13,565 (91.0%)	92,648 (92.5%)	< 0.001
**Region of hospital (%)**			
Northeast	3084 (20.7%)	17,078 (17.0%)	< 0.001
Midwest or North Central	3096 (20.8%)	26,541 (26.5%)	
South	6175 (41.4%)	37,210 (37.1%)	
West	2544 (17.1%)	19,344 (19.3%)	
**Died (%)**	115 (0.8%)	194 (0.2%)	< 0.001

LOS: Length of stay, TOTCHE: Total charge

### Patient demographics between the two groups

A notable difference was observed in the occurrence of blood transfusions during hospitalization between the two genders, with a higher proportion of transfusions observed in females (64.1% vs. 35.9%, *P* < 0.001) (Table 2). Overall, patients who required blood transfusions were relatively older (67.4 years vs. 64.7 years, *P* < 0.001). Consistently, the age distribution between the two groups differed, with a higher proportion of patients aged 71 years and older in the blood transfusion group (41.4% vs. 30.2% *P* < 0.001) (Table 2). Meanwhile, a significant difference was detected in races, with the Black occupying slightly larger proportions in the transfusion group.

### Hospital characteristics of two groups

The significantly more cases of TKR requiring blood transfusions were observed in large hospitals than in small hospitals (*P* < 0.001) (Table 2). Patients who received a blood transfusion were less likely to have undergone elective admission compared to those in the non-transfusion group (*P* < 0.001) (Table 2). Additionally, teaching and urban hospitals exhibited a lower incidence of blood transfusion after TKR (*P* < 0.001). In terms of geographical location, in-hospital blood transfusion was more likely in the South and Midwest or North Central regions, while less likely in the Northeast and West (*P* < 0.001) (Table 2).

### Adverse impact of blood transfusion in TKR

The significantly more deaths (at least 3-fold) were observed among patients receiving blood transfusion than those who did not require blood transfusion (0.2% vs. 0.8%; *P* < 0.001) (Table 2). The median length of hospital stay was extended by three days in the presence of blood transfusion (6 days vs. 3 days; *P* < 0.001) (Table 2). Consequently, the occurrence of blood transfusion was associated with an increase in medical expenses. Notably, there was a substantial rise of $34,731 in total hospital charges associated with blood transfusions ($121,039 vs. $86,308, *P* < 0.001) (Table 2).

### Risk factors of blood transfusion for TKR

Logistic regression analysis was utilized to explore factors associated with blood transfusion, revealing the following indicators: advanced age (≥ 71 years, odds ratio [OR] = 1.49; 95% confidence interval [CI] = 1.38–1.61; *P* < 0.001), female (OR = 1.25; CI = 1.21–1.30), black (OR = 1.45; CI = 1.37–1.53), hispanic (OR = 1.380; CI = 1.28–1.47), large hospital (OR = 1.16; CI = 1.11–1.22) (Table 3), iron-deficiency anemia (OR = 2.43; CI = 2.32–2.55), rheumatoid arthritis/collagen vascular diseases (OR = 1.29; CI = 1.20–1.38), chronic blood loss anemia (OR = 2.72; CI = 2.42–3.06), congestive heart failure (OR = 1.40; CI = 1.31–1.50 ), coagulopathy (OR = 1.90; CI = 1.75–2.05), uncomplicated diabetes (OR = 1.15; CI = 1.10–1.20), lymphoma (OR = 2.00; CI = 1.59–2.51), fluid and electrolyte disorders (OR = 1.84; CI = 1.76–1.93), metastatic cancer (OR = 1.99; CI = 1.47–2.68), other neurological disorders (OR = 1.39; CI = 1.25–1.45), paralysis (OR = 1.72; CI = 1.39–2.17), peripheral vascular disorders (OR = 1.31; CI = 1.20–1.44), pulmonary circulation disorders (OR = 1.36; CI = 1.22–1.53), renal failure (OR = 1.45; CI = 1.37–1.54), valvular disease (OR = 1.24; CI = 1.15–1.34), weight loss (OR = 1.96; CI = 1.76–2.17) (Table 4). Interestingly, protective factors included private insurance (OR = 0.83; CI = 0.80–0.88), elective admission (OR = 0.511; CI = 0.49–0.53), teaching hospital (OR = 0.810; CI = 0.78–0.84), urban hospital (OR = 0.90; CI = 0.83–0.96), hospital in the Midwest or North Central (OR = 0.63; CI = 0.59–0.66), South (OR = 0.87; CI = 0.86–0.91), West (OR = 0.72; CI = 0.68–0.76) (Table 3) regions, and Obesity (OR = 0.81; CI = 0.78–0.84) (Table 4).

**Table 3:** Risk factors associated with blood transfusion after total knee revision

Variable		Multivariate logistic regression		
		OR	95% CI	*P*
Age group (%)				
**≤ 50**		Ref	——	——
**51–60**		0.994	0.920–1.074	0.888
**61–70**		1.082	1.002–1.168	0.043
**≥ 71**		1.490	1.377–1.613	<0.001
**Female**		1.254	1.209–1.302	<0.001
**Race**				
	White	Ref	——	——
	Black	1.449	1.373–1.529	<0.001
	Hispanic	1.380	1.281–1.486	<0.001
	Asian or Pacific Islander	1.282	1.058–1.552	0.011
	Native American	1.040	0.818–1.321	0.751
	Other	1.066	0.995–1.143	0.068
**Elixhauser score**				
	0	Ref	——	——
	1	1.212	1.111–1.323	<0.001
	≥ 2	2.293	2.124–2.475	<0.001
**Type of insurance**				
	Medicare	Ref	——	——
	Medicaid	0.996	0.916–1.083	0.925
	Private insurance	0.834	0.795–0.875	<0.001
	Self-pay	0.929	0.730–1.181	0.548
	No charge	1.407	0.750–2.641	0.288
	Other	0.691	0.625–0.764	<0.001
**Bed size of hospital**				
	Small	Ref	——	——
	Medium	1.110	1.054–1.168	<0.001
	Large	1.162	1.111–1.217	<0.001
**Elective admission**		0.511	0.491–0.532	<0.001
**Teaching hospital**		0.810	0.778–0.842	<0.001
**Urban hospital**		0.895	0.836–0.958	= 0.001
**Region of hospital**				
	Northeast	Ref	——	——
	Midwest or North Central	0.625	0.591–0.661	<0.001
	South	0.866	0.825–0.909	<0.001
	West	0.718	0.677–0.761	<0.001

OR: Odds ratio, CI: Confidence interval

**Table 4:** Relationship between blood transfusion and preoperative comorbidities

Comorbidities		Univariate analysis			Multivariate logistic regression			
		No transfusion	Transfusion	*P*		OR	95% CI	*P*
Preoperative comorbidities								
	Acquired immune deficiency syndrome	120 (0.1%)	24 (0.2%)	0.183		1.208	0.767–1.903	0.416
	Alcohol abuse	1283 (1.3%)	290 (1.9%)	<0.001		1.078	0.940–1.237	0.284
	Iron deficient anemia	8283(8.3%)	3277 (22.0%)	<0.001		2.433	2.320–2.551	<0.001
	Rheumatoid arthritis/collagen vascular diseases	5725 (5.7%)	1262 (8.5%)	<0.001		1.287	1.204–1.376	<0.001
	Chronic blood loss anemia	1008 (1.0%)	439 (2.9%)	<0.001		2.722	2.418–3.063	<0.001
	Congestive heart failure	5042 (5.0%)	1619 (10.9%)	<0.001		1.402	1.312–1.497	<0.001
	Chronic pulmonary disease	18,961 (18.9%)	3113 (20.9%)	<0.001		0.945	0.903–0.989	0.015
	Coagulopathy	2798 (2.8%)	1165 (7.8%)	<0.001		1.895	1.754–2.047	<0.001
	Depression	17,718 (17.7%)	2892 (19.4%)	<0.001		0.991	9.45–1.038	0.693
	Diabetes, uncomplicated	19,239 (19.2%)	3511(23.6%)	<0.001		1.151	1.100-1.203	<0.001
	Diabetes with chronic complications	5747 (5.7%)	1263 (8.5%)	<0.001		1.010	0.941–1.085	0.078
	Drug abuse	1554 (1.6%)	311 (2.1%)	<0.001		1.079	0.948–1.229	0.249
	Hypertension	67,080 (67.0%)	10,927 (73.3%)	<0.001		0.981	0.936–1.028	0.415
	Hypothyroidism	16,547 (16.5%)	2870 (19.3%)	<0.001		1.035	0.988–1.085	0.144
	Liver disease	2448 (2.4%)	583 (3.9%)	<0.001		1.033	0.933–1.142	0.534
	Lymphoma	292 (0.3%)	118 (0.8%)	<0.001		1.998	1.593–2.506	<0.001
	Fluid and electrolyte disorders	10,306 (10.3%)	3555 (23.9%)	<0.001		1.843	1.758–1.931	<0.001
	Metastatic cancer	156 (0.2%)	73 (0.5%)	<0.001		1.986	1.473–2.678	<0.001
	Other neurological disorders	3877 (3.9%)	1061 (7.1%)	<0.001		1.348	1.252–1.452	<0.001
	Obesity	28,611 (28.6%)	4164 (27.9%)	1.22		0.809	0.776–0.843	<0.001
	Paralysis	307 (0.3%)	107 (0.7%)	<0.001		1.715	1.358–2.165	<0.001
	Peripheral vascular disorders	2580 (2.6%)	703 (4.7%)	<0.001		1.311	1.197–1.435	<0.001
	Psychoses	3435 (3.4%)	691 (2.4%)	<0.001		1.153	1.056–1.259	0.002
	Pulmonary circulation disorders	1374 (1.4%)	500 (3.4%)	<0.001		1.364	1.218–1.528	<0.001
	Renal failure	7465 (7.5%)	2344 (15.7%)	<0.001		1.454	1.374–1.539	<0.001
	Solid tumor without metastasis	638 (0.6%)	158 (1.1%)	<0.001		1.228	1.020–1.479	0.003
	Peptic ulcer disease excluding bleeding	192 (0.2%)	27 (0.2%)	0.785		0.84	0.528–1.222	0.307
	Valvular disease	3666 (3.7%)	959 (6.4%)	<0.001		1.239	1.145–1.341	<0.001
	Weight loss	1267 (1.3%)	664 (4.5%)	<0.001		1.955	1.763–2.167	<0.001

OR: Odds ratio, CI: Confidence interval

### Factors associated with blood transfusions during TKR

Blood transfusions occurred more frequently in sepsis, acute myocardial infarction, deep vein thrombosis, convulsion, pulmonary embolism, gastrointestinal bleeding, heart failure, renal insufficiency, pneumonia, respiratory disease, wound infection, lower limb nerve injury, hemorrhage/seroma/hematoma, wound rupture/non healing, urinary tract infection, acute renal failure, postoperative delirium (*P* < 0.001) (Table 5). During multivariate analysis, blood transfusion was associated with sepsis (OR = 1.23; CI = 1.13–1.33), acute myocardial infarction (OR = 1.79; CI = 1.50–2.15), deep vein thrombosis (OR = 1.79; CI = 1.52–2.09), pulmonary embolism (OR = 1.55; CI = 1.24–1.94), gastrointestinal bleeding (OR = 3.13; CI = 2.39–4.10), heart failure (OR = 1.99; CI = 1.85–2.14), renal insufficiency (OR = 3.18; CI = 1.65–6.12), pneumonia (OR = 1.67; CI = 1.45–1.92), wound infection (OR = 1.57; CI = 1.35–1.83), lower limb nerve injury (OR = 1.67; CI = 1.51–1.85), hemorrhage/seroma/hematoma (OR = 2.36; CI = 2.13–2.62), wound rupture/non healing (OR = 1.44; CI = 1.30–1.60), urinary tract infection(OR = 2.09; CI = 1.94–2.26), acute renal failure (OR = 2.33; CI = 2.20–2.48), and postoperative delirium (OR = 1.85; CI = 1.61–2.14).

**Table 5:** Relationship between blood transfusion and postoperative complications

Complications		Univariate analysis				Multivariate logistic regression		
		No transfusion		Transfusion	*P*	OR	95% CI	*P*
Medical complications								
	Sepsis	3254 (3.2%)	1137 (7.6%)		<0.001	1.225	1.130–1.329	<0.001
	Acute myocardial infarction	435 (0.4%)	211 (1.4%)		<0.001	1.792	1.497–2.146	<0.001
	Deep vein thrombosis	583 (0.6%)	267 (1.8%)		<0.001	1.785	1.523–2.094	<0.001
	Convulsion	349 (0.3%)	80 (0.5%)		<0.001	1.453	1.131–1.869	0.004
	Pulmonary embolism	321 (0.3%)	128 (0.9%)		<0.001	1.550	1.239–1.939	<0.001
	Gastrointestinal bleeding	134 (0.1%)	115 (0.8%)		<0.001	3.130	2.389–4.099	<0.001
	Heart failure	3369 (3.4%)	1263 (8.5%)		<0.001	1.990	1.853–2.137	<0.001
	Renal insufficiency	23 (0.0%)	17 (0.1%)		<0.001	3.175	1.647–6.122	0.001
	Pneumonia	711 (0.7%)	374 (2.5%)		<0.001	1.666	1.447–1.918	<0.001
	Respiratory disease	281 (0.3%)	91 (0.6%)		<0.001	1.339	1.039–1.726	0.024
	Urinary tract infection	2635 (2.6%)	1097 (7.4%)		<0.001	2.093	1.937–2.261	<0.001
	Acute renal failure	5179 (5.2%)	2241 (5%)		<0.001	2.333	2.199–2.475	<0.001
	Postoperative delirium	746 (0.7%)	319(2.1%)		<0.001	1.853	1.608–2.135	<0.001
**Surgical complications**								
	Wound infection	740 (0.7%)	243 (1.6%)		<0.001	1.572	1.346–1.835	<0.001
	Lower limb nerve injury	2011 (2.0%)	507 (3.4%)		<0.001	1.672	1.511–1.850	<0.001
	Hemorrhage/seroma/hematota	1416 (1.4%)	582 (3.9%)		<0.001	2.360	2.130–2.616	<0.001
	Wound rupture/non healing	2036 (2.0%)	553 (3.7%)		<0.001	1.442	1.303–1.595	<0.001

OR: Odds ratio, CI: Confidence interval

## Discussion

This investigation presents a comprehensive health-economic analysis of patients undergoing in-hospital blood transfusion following total knee revision. Because of an increased focus on enhancing surgical techniques, component design, and the implementation of strict transfusion guidelines, clinical protocols for maintaining minimum preoperative Hb levels, as well as the implementation of new drugs, hemostatics and devices for hemostatics [15–20], there was a gradual decrease in blood transfusion rates from 2010 to 2019 (from 20.4 to 6.5%) (Fig. 2). The overall infection rate after TKA was 1.1% at 3 months and 1.6% at 1 year [21]; increased infections were associated with increased TKR rates. Effective management of TKR typically involves debridement, with or without implant removal, resulting in a complex and prolonged procedure and extended hospital stays [22]. In comparison to TKA, the transfusion rate in TKR is higher. Therefore, understanding the risk factors for transfusion in TKR is crucial for effective control [23, 24].

**Fig. 3:**
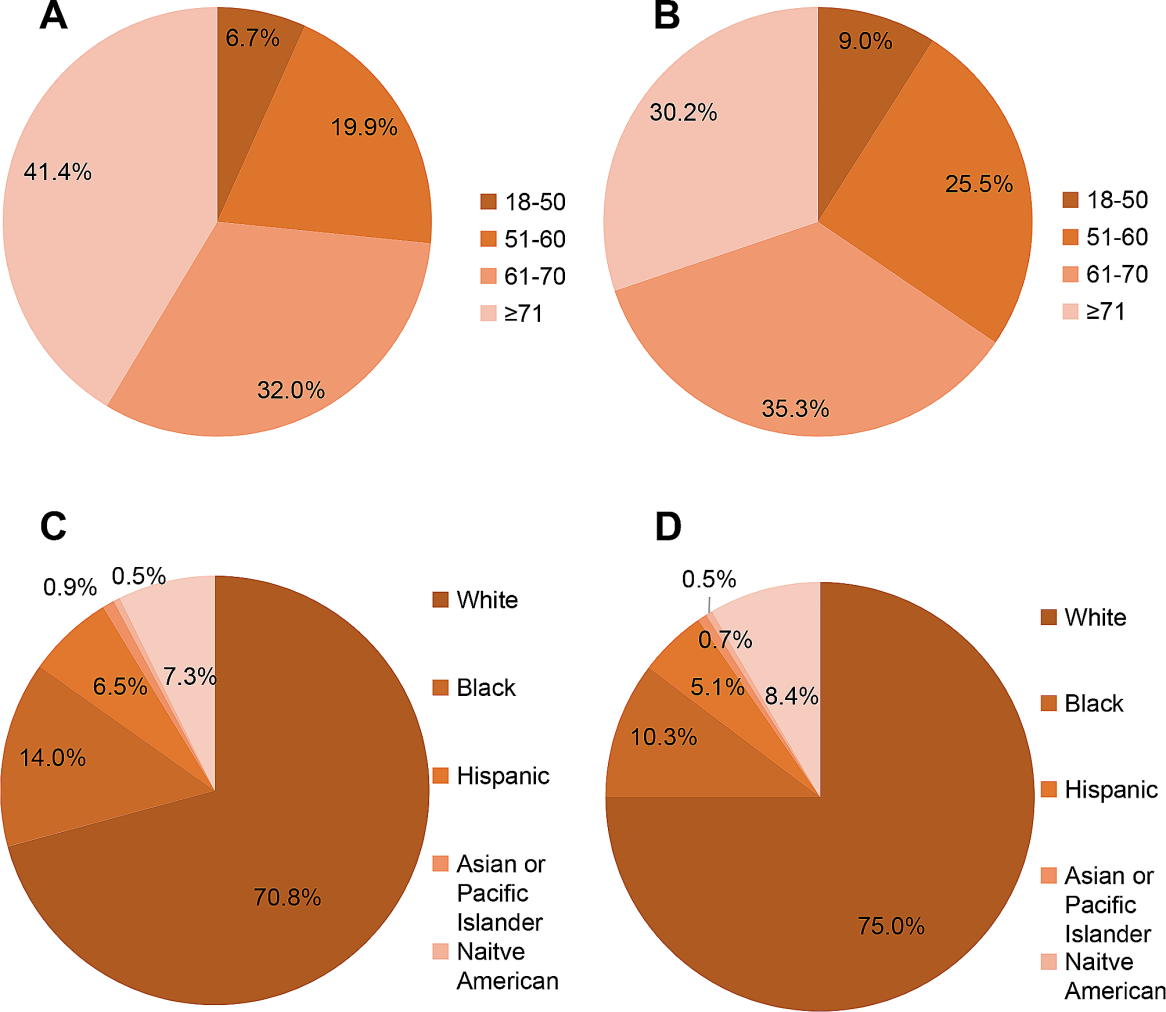
Patient demographics between the two surgical groups. **A**: Age distribution analysis of blood transfusion patients. **B**: Analysis of age distribution of patients without blood transfusion. **C**: Racial distribution analysis of blood transfusion patients. **D**: Racial distribution analysis of patients without blood transfusion

There is an increasing consensus suggesting that both older age and being female are linked to increased blood transfusion risk in the context of total knee revision [24–26]. In our investigation, older age (≥ 71 years) and being female emerged as independent risk factors for blood transfusion (Fig. 3A and B). This correlation may be attributed to older individuals having a higher prevalence of comorbidities and weakened hematopoietic function [25, 27]. Additionally, women are more prone to anemia, experience longer postoperative recovery cycles, and are at a heightened risk of complications [28]. During logistic regression analysis, compared to White individuals, the Black and Hispanic have a higher incidence of blood transfusions following TKR (Fig. 3C and D). Both Black and Hispanic patients have been shown to have lower levels of health literacy. Complex factors contribute to disparities, including diminished access to care that results in delayed patient presentations, substandard care, impaired clinician-patient communication, and insufficient post-hospital support systems [29]. Conversely, blood transfusions were less frequent in Asians or Pacific Islanders. This disparity may be attributed to higher rates of anemia, lower mean corpuscular volume, and lower serum transferrin saturation in this population, suggesting that racial or genetic heterogeneity might contribute to the likelihood of requiring blood transfusion [30, 31].

**Fig. 4:**
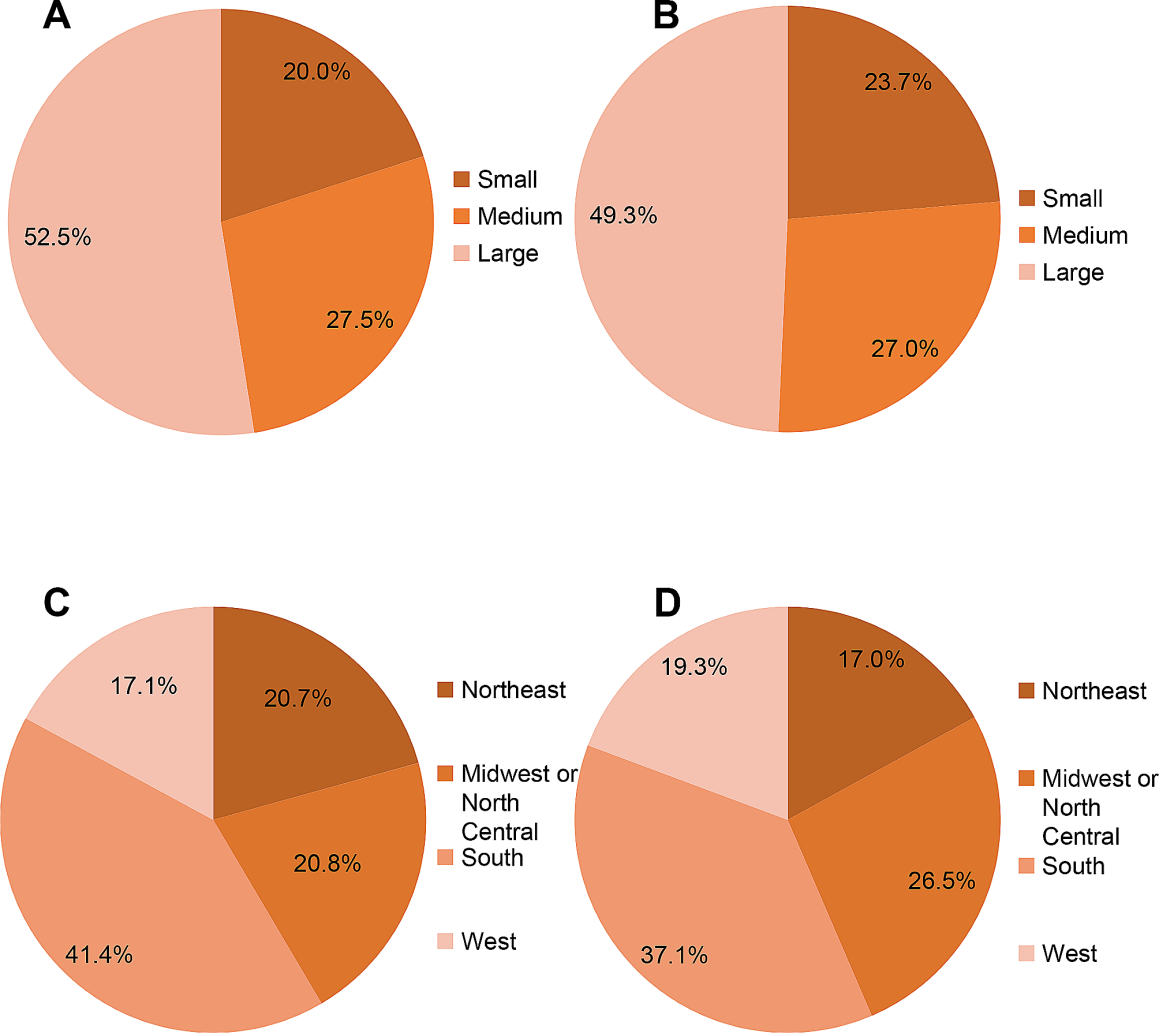
Hospital characteristics between the two surgical groups. **A**: Analysis of the number of hospital beds for blood transfusion patients. **B**: Analysis of the number of hospital beds for patients without blood transfusion. **C**: Analysis of hospital regional distribution of blood transfusion patients. **D**: Analysis of hospital regional distribution of patients without blood transfusion

The literature has consistently reported that blood transfusion in the context of TKR is associated with extended length of stay, increased medical costs, and higher mortality rates [21, 32]. Consistent outcomes were found in the present study (Table 2). In the presence of blood transfusion, the median length of stay was extended by 3 days, and the total hospital charge per admission increased by $34,731, this may be due to blood transfusion itself, LOS and complications associated with blood transfusion [33–35]. Patients undergoing TKR via elective admission were less likely to experience blood transfusion, possibly because most elective cases involve individuals with relatively healthier conditions or adequate pre-operative preparations, while emergent cases tend to be more severe [36]. The likelihood of blood transfusion was higher in large hospitals but lower in small hospitals (Fig. 4A, B), potentially related to the complexity of surgeries and the higher volume of surgical patients [24]. In addition, teaching and urban hospitals emerged as protective factors against blood transfusion, possibly due to their standardized blood management practices, the use of drugs and equipment for bleeding control and blood management, and the skilled surgical techniques employed by their surgeons [37]. In-hospital mortality was three times higher in patients affected by blood transfusion compared to those unaffected. Regarding the hospital’s region, the Midwest or North Central, South, and West regions correlated with lower blood transfusion rates after TKR (Fig. 4C and D). However, the reasons for this remain unclear and are likely multifactorial [35].

Numerous studies focusing on blood transfusion following TKA have emphasized the importance of pre-screening, risk stratification, and appropriate management to enhance outcomes [24, 38]. Consequently, to prevent the need for blood transfusion, it is crucial to comprehensively understand the preoperative risk factors. The utilization of logistic regression in our study yielded results consistent with prior research. As anticipated, iron deficient anemia and chronic blood loss anemia were identified as factors that can elevate the risk of blood transfusion, possibly linked to lower preoperative hemoglobin levels in patients [37, 39]. Maempel et al. [40] revealed that undergoing total knee arthroplasty were associated with a 6-fold increase in transfusion rates. However, patients with coagulopathy may experience significant blood loss during TKR, potentially leading to the necessity for blood transfusion [41].

**Fig. 5:**
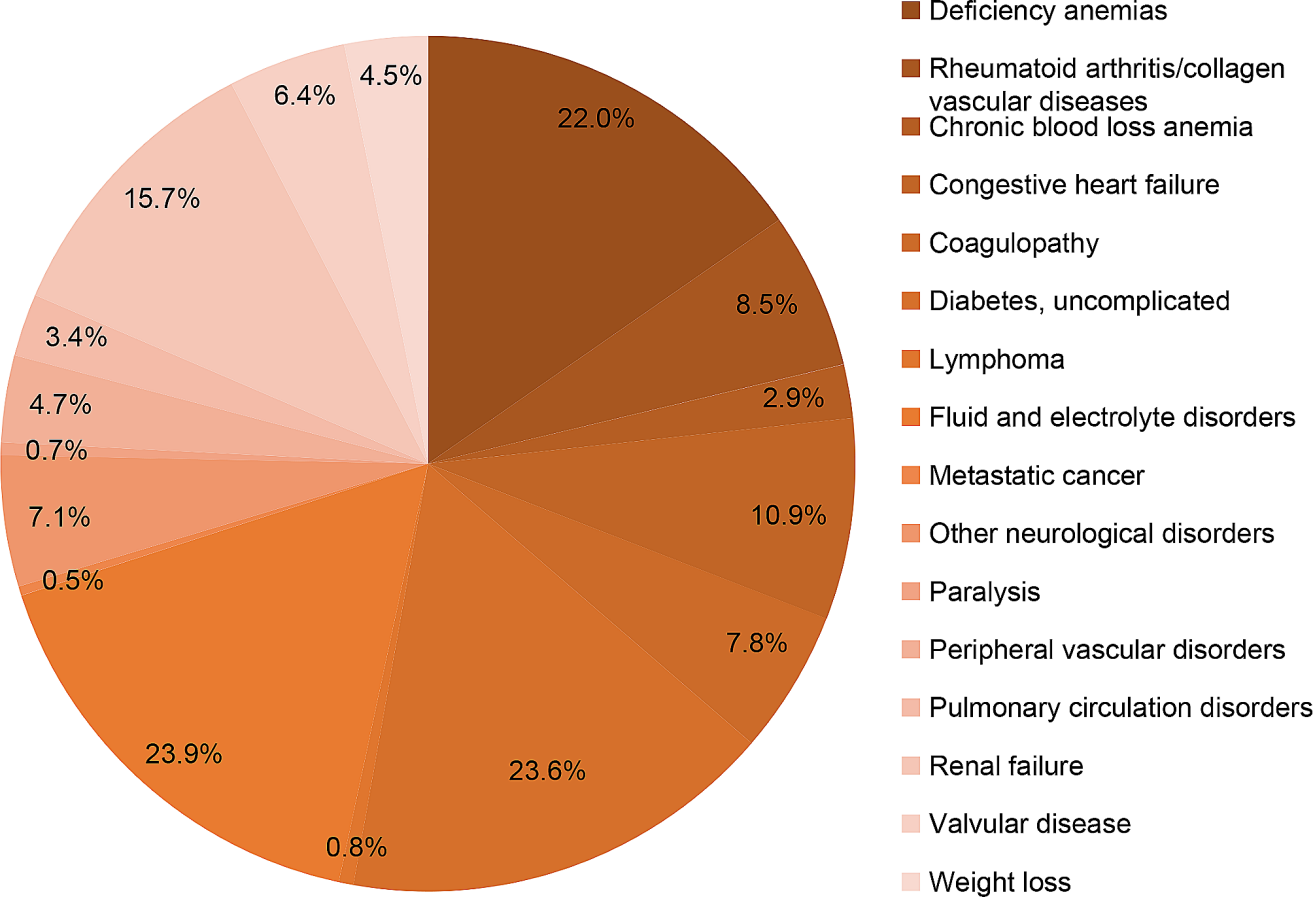
Incidence of preoperative comorbidities related to blood transfusion

Patient-related factors associated with blood transfusion in our study encompassed rheumatoid arthritis (RA)/collagen vascular diseases and uncomplicated diabetes (Fig. 5), both of which are systemic autoimmune disorders. This association may be attributed to the heightened susceptibility of these patients to preoperative anemia and low protein levels [42]. Congestive heart failure and valvular disease, known for inducing organ hypoxia, ischemia, and inadequate blood volume, were identified as factors increasing the likelihood of blood transfusion. However, the underlying cause of this relationship remains unknown. In our investigation, risk factors for blood transfusion included lymphoma, metastatic cancer, and weight loss (Fig. 5), indicating a relatively poor health condition that might be further exacerbated by intraoperative blood loss [43].

**Fig. 6:**
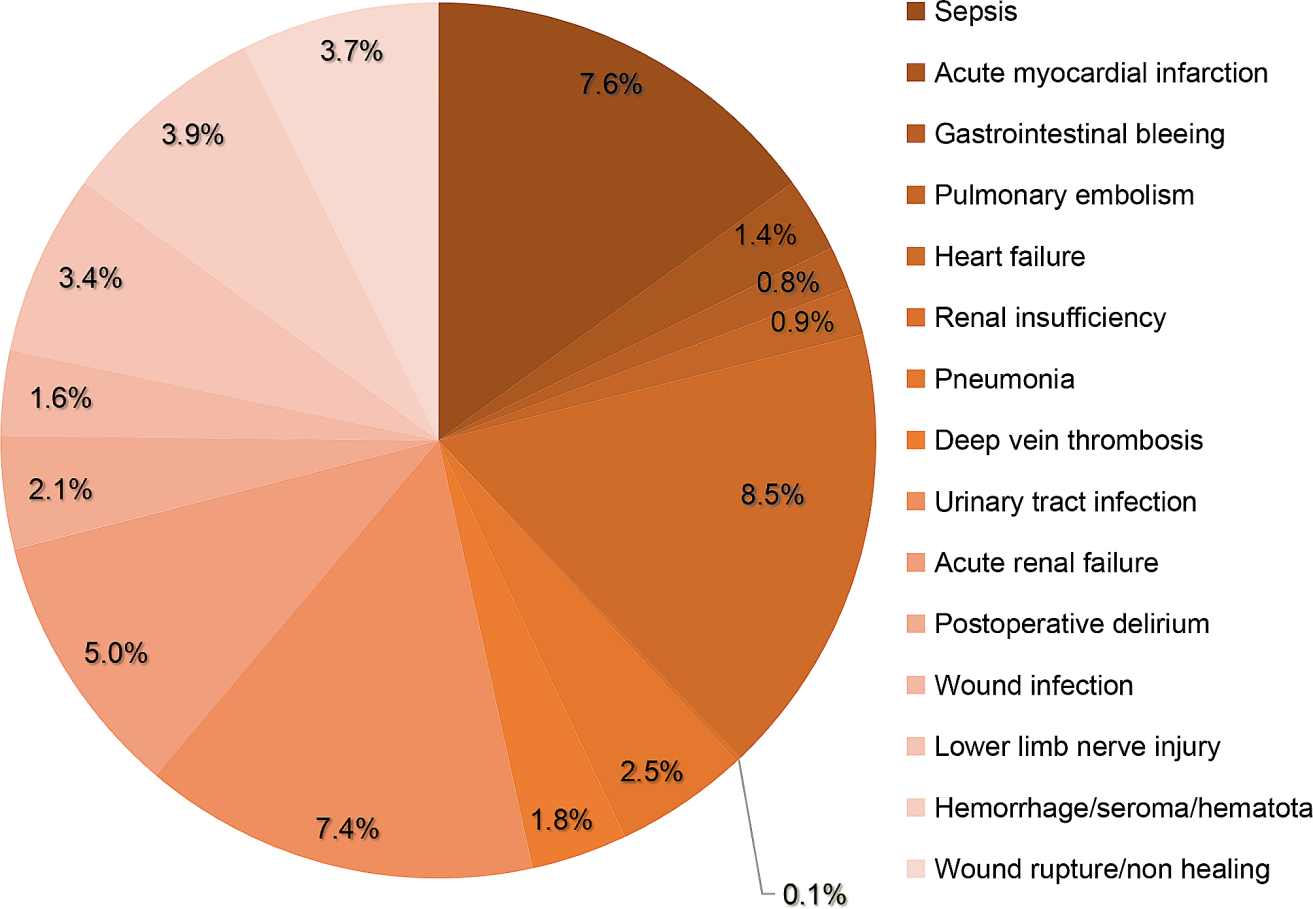
Incidence of postoperative complications related to blood transfusion

Our results indicate that sepsis, acute myocardial infarction, deep vein thrombosis, pulmonary embolism, gastrointestinal bleeding, heart failure, renal insufficiency, pneumonia, urinary tract infection, acute renal failure, postoperative delirium, wound infection, lower limb nerve injury, hemorrhage/seroma/hematoma, wound rupture/non-healing were linked with blood transfusion (Fig. 6).

Consistent with the literature, our findings indicate that blood transfusion after TKR was associated with venous thromboembolism and pneumonia [44]. In TKR, patients experience loss of whole blood but are transfused with concentrated blood, potentially contributing to the higher viscosity of the transfused blood. Another explanation could be that the transfusion of stored erythrocytes itself influences the coagulative process [45]. It is widely thought that transfusion of red blood cell units stored for more than 20 days increases the venous thromboembolism risk [46]. Stored red blood cell units have been associated with reduced nitric oxide levels, leading to vasoconstriction, elevated lactate levels causing a decrease in pH, and the release of proinflammatory factors, all of which can impact coagulation and enhance hypercoagulability [47, 48].

Our study revealed that blood transfusion after TKR was associated with perioperative infectious complications including sepsis, wound infection, wound rupture/non-healing, urinary tract infection and pneumonia. In line with the findings of prior research, adverse effects occurred in blood transfusions [49, 50]. The effect of transfusions may have been in part related to low-dose bacterial contamination from the phlebotomy site and methods of blood handling and storage [51]. According to clinical experience, the infection patients are more likely to receive blood transfusions. Probably because infection with greater circulatory dysfunction, greater susceptibility to anemia and blood transfusions [52].Post-blood transfusion, there is an increased likelihood of renal insufficiency and acute renal failure, potentially stemming from that blood transfusions may increase the amount of circulating free iron and exacerbate the stress injury during surgery, which associated with renal organ damage [53]. However, red blood cell production and erythrocyte lifespan may be diminished in acute renal failure, resulting in anaemia and blood transfusion [54]. Additionally, blood transfusions was associated with acute myocardial infarction and heart failure, possibly due to changes in blood viscosity leading to an increased circulating blood volume and subsequent cardiac strain [55].And in patients with acute myocardial infarction, sustaining an elevated haemoglobin level may enhance clinical outcomes, potentially leading to transfusions, by enhancing oxygen delivery to the susceptible myocardium [45].The blood transfusions and complications that is an association and does not prove causality. The inference of causality between blood transfusion and adverse outcomes remains difficult due to methodological limitations of observational and database studies. Transfused patients are likely to have a higher degree of comorbidity, or postoperative complications, which may be the cause of the adverse outcome rather than the transfusion itself. Despite attempts to control for this confounding, causation cannot be proved. Thus, further validation through prospective studies is still needed for the given results [35].”

Our study has several limitations. Firstly, patient information was recorded only up to the point of discharge, suggesting that complications occurring after discharge are not recorded in the Nationwide Inpatient Sample database. Secondly, being a retrospective observational study, our investigation shares the limitations inherent in such designs. The NIS database lacks specific details, such as the volume of blood loss, pre-, peri-, postoperative hemoglobin values, or the number of units of blood transfused [7] and using of hemostatics. These limitations highlight that our results should be interpreted with caution, given that only data available in the NIS database could be analyzed. Indeed, certain well-established risk factors were not accessible in the Nationwide Inpatient Sample database, including details such as the type of anesthesia, duration of the operation, commonly used perioperative medications (opioids, benzodiazepines, and ketamine), sedation during anesthesia recovery, and functional impairment. Additionally, administrative data tends to exhibit high specificity (low false-positive rate) but low sensitivity (high false-negative rate) in identifying adverse events. This characteristic may lead to an underestimation of the incidence of blood transfusion following total knee revision [27].

## Conclusion

Our findings indicate that the incidence of blood transfusion in TKR continued to decrease with an overall incidence of 13.0% between 2010 and 2019. Notwithstanding advancements, transfusions continue to transpire, with a higher incidence observed in female patients, older patients (≥ 71), patients afflicted with particular situation (e.g., iron deficiency anemia, chronic blood loss anemia, lymphoma, metastatic cancer, weight loss). Nevertheless, obesity was recognized as a protective factor. Additionally, gastrointestinal bleeding, renal insufficiency, urinary tract infection, hemorrhage/seroma/hematoma, and acute renal failure were all linked to blood transfusions. There is an association between blood transfusions and increased hospital costs and duration of stay. It is recommended that comprehensive preoperative assessment and medical optimization be performed on these patients, in addition to preoperative anemia management strategies, in order to reduce the likelihood of perioperative transfusion.

### Electronic supplementary material

Below is the link to the electronic supplementary material.

Supplementary Material 1

## Data Availability

The datasets are available at https://www.ahrq.gov/data/hcup/index.html.

## Abbreviations

Total knee arthroplasty: TKA
Total knee revision: TKR
Length of stay: LOS
Total joint arthroplasty: TJA
Nationwide inpatient sample: NIS
International classification of diseases: ICD
Figure: Fig
Statistical package for the social sciences: SPSS
Odds ratios: OR
Confidence intervals: CI
Rheumatoid arthritis: RA
Acquired immunodeficiency syndrome: AIDS
